# Interaction of PM_2.5_ and pre-pregnancy body mass index on birth weight: A nationwide prospective cohort study

**DOI:** 10.3389/fendo.2022.963827

**Published:** 2022-07-26

**Authors:** Hanze Du, Yuxin Sun, Yuelun Zhang, Shirui Wang, Huijuan Zhu, Shi Chen, Hui Pan

**Affiliations:** ^1^ Department of Endocrinology, Key Laboratory of Endocrinology of National Health Commission, Translation Medicine Centre, Peking Union Medical College Hospital, Peking Union Medical College, Chinese Academy of Medical Sciences, Beijing, China; ^2^ Eight-Year Program of Clinical Medicine, Peking Union Medical College, Chinese Academy of Medical Sciences and Peking Union Medical College, Beijing, China; ^3^ Medical Research Center, Peking Union Medical College Hospital, Chinese Academy of Medical Sciences and Peking Union Medical College, Beijing, China; ^4^ Key Laboratory of Endocrinology of National Health Commission, Department of Endocrinology, State Key Laboratory of Complex Severe and Rare Diseases, Peking Union Medical College Hospital, Chinese Academy of Medical Science and Peking Union Medical College, Beijing, China

**Keywords:** air pollution, birthweight, negative interaction, metabolic status, obesity, fine particulate matter (PM2.5), pre-pregnancy body mass index

## Abstract

**Background:**

Fine particulate matter (PM_2.5_), one of the most common air pollutants worldwide, has been associated with many adverse birth outcomes in some studies. Pre-pregnancy body mass index (BMI) is an important indicator of maternal obesity that may also contribute to a wide range of birthweight outcomes. Both PM_2.5_ and maternal obesity have been found associated with issues on neonatal birthweight respectively, and more attentions and interests are focusing on their combined effect on pregnancy outcomes.

**Purpose:**

To explore the modifying effect of pre-pregnancy BMI on the association between gestational PM_2.5_ and birthweight; to investigate the interactive effect between gestational PM_2.5_ and pre-pregnancy BMI on birthweight among pregnant women during three trimesters and the whole pregnancy.

**Methods:**

This nationwide cohort study used the National Free Preconception Health Examination Project (NFPHEP) data collected from January 1, 2010, to December 31, 2012. A total population of 248,501 Chinese women from 220 counties registered this project. Pre-pregnancy BMI as a common anthropometric examination was collected during preconception investigation, and gestational PM_2.5_ was derived from a hindcast model for historical PM_2.5_ estimation from satellite-retrieved aerosol optic depth. Subgroup analysis was conducted to explore a potential modifying effect on the association between PM_2.5_ and birthweight during pregnancy by four pre-pregnancy BMI subgroups. Interaction analysis by introducing product terms to multivariable linear regression was also used to examine whether there was an interactive relationship between PM_2.5_ and pre-pregnancy BMI.

**Results:**

Totally, 193,461 participants were included in our study. The average concentration of PM_2.5_ was 75.33 μg/m^3^. Higher exposure of PM_2.5_ during the entire pregnancy was associated with higher birthweight (17.15 g per 10 μg/m3; 95% CI:16.15, 18.17). Each 10 μg/m^3^ increase in PM_2.5_ during the first, second, and third trimesters was associated with increases in birthweight by 14.93 g (95%CI: 13.96, 15.89), 13.75 g (95% CI: 12.81, 14.69), and 8.79 g (95% CI: 8.09, 9.49), respectively. Higher pre-pregnancy BMI per kg/m^2^ was associated with an increase of birthweight by 7.012 g (95% CI: 6.121, 7.902). Product terms between PM_2.5_ and pre-pregnancy BMI were significant for the first, second trimesters, and the entire duration of pregnancy.

**Conclusions:**

Our results found both gestational PM_2.5_ exposure and pre-pregnancy BMI respectively correlated with the increase of birthweight. A negative interaction between pre-pregnancy BMI and gestational PM_2.5_ was discovered in term of birthweight gain. Avoidance of high-dose exposure to PM_2.5_ during the early and middle stages of pregnancy and pre-pregnancy overweight/obesity may help prevent high birthweight.

## Introduction

Birthweight is an important determinant of both maternal and neonatal health as well as lifelong well-being. High birthweight has been proved to be strongly correlated with health conditions such as cardiovascular diseases, obesity, type 2 diabetes mellitus (1, 2). High birthweight coupled with postnatal growth may increase the early presence of cardiometabolic risk factors and vascular imprinting (3). Studies have showed that the increased prevalence of high birthweight or large for gestational age (LGA) becomes a worldwide health issue (4). The prevalence of macrosomia in China was 6.9% in 2007-2008, and ranged from 7.3% to 8.7% in 2010-2014, higher than the average of 23 low-income and middle-income countries (5, 6), while the incidence of low birthweight in babies (≥28 gestational weeks) was unchanged between 2012-2018 at around 5.5% (5). Birthweight is closely related to intrauterine environment, whose establishment is rooted in multi-factorial interactions including maternal metabolic status, genetic expression, life style, and also physical environment (7–9).

Recently, studies on the combined effect of metabolism and environmental factors on health have increased, of which maternal obesity and air pollution such as PM_2.5_ attract a lot of attention (10, 11). Obesity has emerged as a major issue. From 1995 to 2014, the prevalence of overweight raised from 4.2% to 14.0% and the obesity rate from 1.0% to 6.4% (5). Pre-pregnancy BMI, as an acceptable indicator of metabolic gradations of thinness and fatness, has profound effects on both mothers and fetuses by influencing the intrauterine environment. Pre-pregnancy overweight/obesity was found associated with higher risks of maternal gestational diabetes mellitus, pre-eclampsia, post-delivery weight retention and dysglycemia (12–14). Adverse outcomes on fetus due to pre-gestational overweight/obese could be preterm birth, stillbirth, caesarean delivery and LGA (15, 16). As for the environmental factors, adverse impacts of PM_2.5_ on public health have been huge concerns worldwide, especially in developing countries. However, studies on the adverse effects of gestational PM_2.5_ exposure on birthweight can be controversial and inconsistent (17, 18). Majority of studies demonstrated correlations between PM_2.5_ and low birthweight or small for gestational age (SGA) (19–25), while some studies confirmed associations between PM2.5 and LGA or macrosomia (10, 17, 18, 26). Previous studies pointed out that marked differences on PM_2.5_ pollution between rapidly developing and developed countries might cause inconsistency in results (17, 18). Air pollution was an extremely serious issue of China during 2010s, when many provinces and cities had annual average PM2.5 over 80 μg/m^3^ (18). A recent study showed that the five most polluted megacities (Delhi, Cairo, Xi’an, Tianjin and Chengdu) all had an annual average concentration of PM_2.5_ greater than 89 μg/m^3^ in 2013, while PM_2.5_ in many other countries could be much lower (23, 24, 27, 28). Race is another difference between countries and studies exemplified by the fact that the Asian prevalence of overweight/obesity is far less than Caucasian (29). Studies on the combination effect between gestational weight gain (GWG) and air pollutions such as PM_2.5_ have reported similar interactions on birth outcomes, and we hope to take further steps in exploring a potential interaction of Chinese population between maternal obesity and air pollutions on birthweight.

Our study was based on a large national cohort during 2010s when air pollution was extremely serious in China, compared to previously reported studies in developed countries. Combining both maternal metabolic status and atmospheric environment, it is necessary to explore their effects on birthweight respectively and jointly. We aimed to explore whether higher dose of PM_2.5_ exposure during pregnancy was associated with growth in birthweight nationwide in China; to explore a potential interaction effect between pre-pregnancy BMI and PM_2.5_ on birthweight.

## Materials and methods

### Study design and population

Data for this national cohort study were derived from the National Free Preconception Health Examination Project (NFPHEP) operated by the National Research Institute for Family Planning and conducted in 220 counties from 31 provinces and province-level municipalities. The project was launched by the National Health Commission of China, lasted for 3 years from January 1, 2010, to December 31, 2012, with the aim to provide free preconception health examinations and follow-up of pregnancy outcomes for married couples planning a pregnancy within next 6 months. The health examinations were conducted by trained and qualified staff and the data was collected in a face-to-face way of investigation, including 371 items in total such as maternal medical history, contraception measures, familial disorders, physical and laboratory examinations, life style and demographic characteristics of parents as well as neonatal birth outcomes. Other detailed information on design, organization and implementation of this project were recorded elsewhere (30–32). The study was approved by the institutional review board of the National Research Institute for Family Planning, Beijing, China and received formal consent from the participants.

### Outcome and exposure assessment

The outcome of our study was birthweight in continuous values recorded by information recorders. Infants with implausible birthweights (≤500 g or ≥5,000 g) were excluded for sensitivity analysis. PM_2.5_ concentrations in 31 provinces were obtained from the Chinese Center for Disease Control and Prevention estimating historical PM_2.5_ concentrations in China from satellite data by using an ensemble machine-learning model (33). The missing satellite data were filled by multiple imputation. The modeling domain was divided into seven regions using a spatial clustering method, and a set of machine learning models were trained in each region separately. A spatial cluster-based model was expected to capture the spatiotemporal variation in PM_2.5_ more accurately by controlling unobserved spatial heterogeneity. To put it simple, the machine-learning model was composed of two parts: three prediction models and the ensemble model. The prediction models for PM_2.5_ concentrations included generalized additive model, random forest model and extreme gradient boosting model, and the ensemble model was an ensemble GAM model combining the three individual models, which could obtain a spatially continuous prediction surface. The model was trained by satellite data and PM_2.5_ ground monitoring records at 1,593 monitoring stations across mainland China from 2013 to 2016 and data during the first months of 2017 was applied for hindcast evaluation. PM_2.5_ concentrations in 2008 in Beijing was also obtained to evaluate the model. The final ensemble prediction characterized the spatiotemporal distribution of daily PM_2.5_ well with the cross-validation (CV) *R^2^* 0.76, root mean square error, RMSE 16 μg/m^3^. Daily county-specific PM_2.5_ concentrations were collected and calculated in the form of trimester-specific mean values, in which first trimester refers to 1-3 months’ gestation, second trimester refers to 4-6 months’ gestation, and the third trimester refers to the rest months of gestation. In our study, subjects who moved during the follow-up were excluded to ensure the homogeneity of prenatal living environment. Pre-pregnancy BMI was segmented into four groups (BMI<18.5 kg/m^2^, BMI 18.5-23.9 kg/m^2^, BMI 24.0-28.0 kg/m^2^, BMI>28.0 kg/m^2^), corresponding to four distinct BMI levels, “underweight”, “normal”, “overweight” and “obese” respectively (31).

### Referred variables

The variables selected and analyzed in our study were maternal and neonatal characteristics such as neonate’s sex, maternal age, gestational week, educational level, maternal smoking or alcohol intake during pregnancy, multiparity, pre-pregnancy diabetes mellitus, pre-pregnancy hypertension, birth weight, the season of delivery.

### Statistical analysis

Unadjusted and adjusted linear regressions were used to evaluate the associations between trimester-specific PM_2.5_ concentrations and neonatal birthweight as well as the association between pre-pregnancy BMI and birthweight. The confounding factors for adjustment included maternal age at delivery, neonatal sex (male, female), smoking or alcohol intake during pregnancy (still, quit, never), gestational week, maternal educational level (junior high school, senior high school, college), prolonged pregnancy (yes, no), multiparity (yes, no), pre-pregnancy diabetes mellitus (yes, no), and pre-pregnancy hypertension (yes, no) (17, 18). The dose-response relationships between PM_2.5_ concentration and birthweight were further investigated using restricted cubic spline models (node was 4). Subgroup analysis was conducted in four BMI intervals (“underweight”, “normal”, “overweight” and “obese”) to explore whether the dose-response relations between PM_2.5_ and birthweight would change under different BMI levels. Segments of PM_2.5_ concentrations used for calculating the average birthweights for each segment were conducted based on the IAQI (individual air quality index) recommended by WHO, which were 0-35 μg/m^3^ (Chinese guideline II), 35-75 μg/m^3^ (mild), 75-115 μg/m^3^ (moderate), 115-150 μg/m^3^ (severe) (34, 35). Multivariable linear regression was conducted involving main effect items, interaction effect item, and other confounding factors for adjustments. Centralized variables were multiplied as an interactive item and added in a regression model. β coefficients and 95% CI were reported. Sensitivity analysis was conducted by excluding some extreme observations in birthweight (≤500 g or ≥5,000 g). Multivariable linear regressions of PM_2.5_ and pre-pregnancy BMI with birthweight (without outliers), and interaction analysis between PM_2.5_ and pre-pregnancy BMI were conducted.

The data cleaning process and descriptive analysis were conducted using SPSS 26.0 (SPSS, Inc., Chicago, IL, USA). Absolute standardized difference was used to check the imbalance of baseline characteristics among different pre-pregnancy BMI subgroups, and a value larger than 0.1 was regarded as baseline imbalance (36). Since absolute standardized difference can only be calculated between two groups, the maximum value among different groups was used. Linear regressions, restricted cubic spline curves, interaction effect analysis and subgroup analysis were all performed using R version 4.1.2 (R Foundation for Statistical Computing, Vienna, Austria), and “foreign”, “rms”, “ggplo2”, “survival”, “MASS”, “splines” packages were applied in our analysis. A two-sided P-value <0.05 was considered statistically significant.

## Results

**Figure 1:**
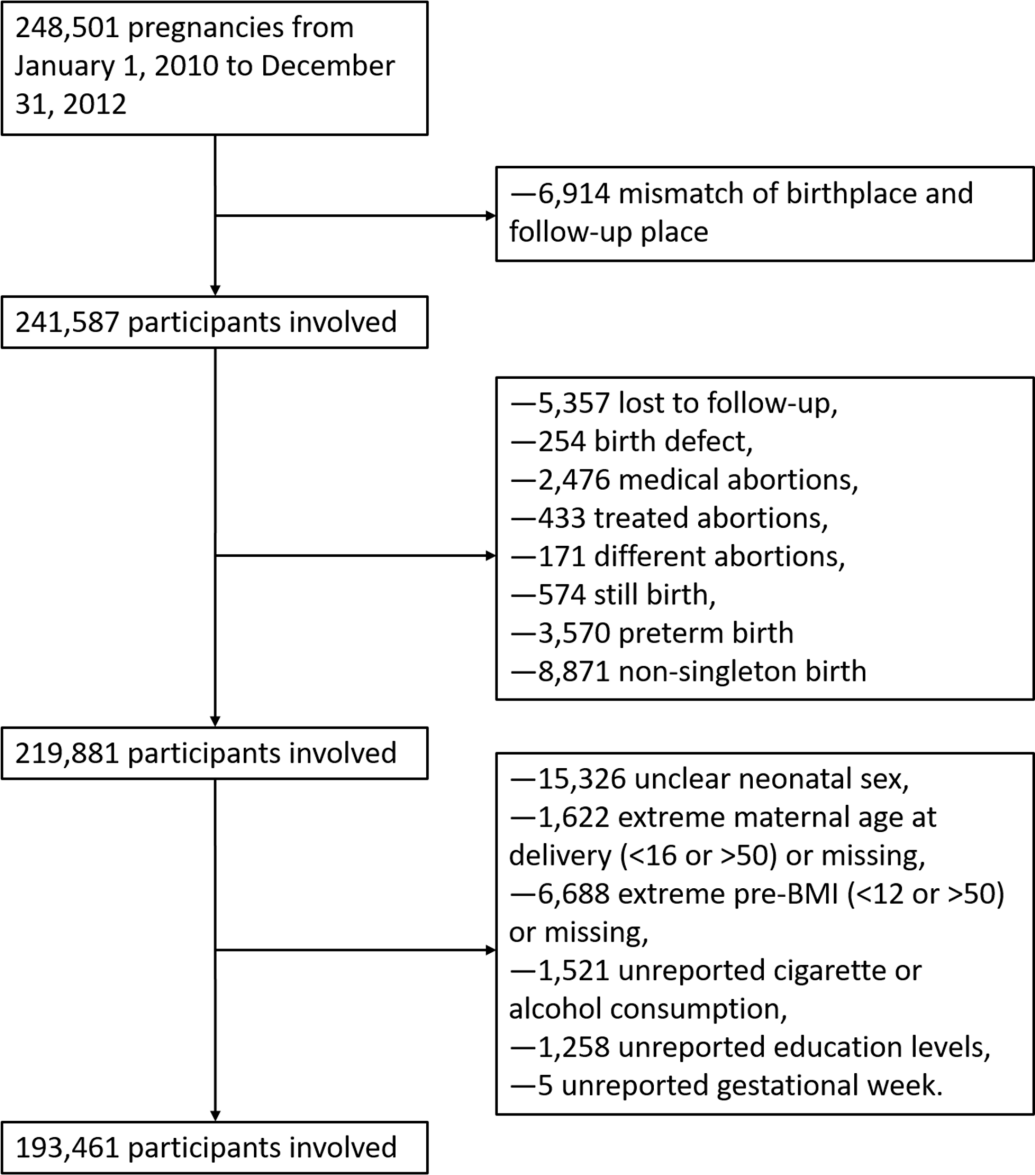
Flowchart of inclusion and exclusion.

The process of inclusion and exclusion of participants was recorded in **Figure 1**. The distribution of PM_2.5_ across the entire pregnancy in China was calculated according to different provinces and recorded in **supplementary materials** (**Table S1**). An initial population of 248,501 participants were preliminarily screened by a standard of exclusion and inclusion in a flowchart (**Figure 1**). The definition of loss to follow-up was that participants had preconception examination but had not received pre-natal or post-natal examination and questionnaires yet by 1 month after the expected data of confinement. Participants whose birthplaces and follow-up places did not match were excluded. 241,587 participants were further screened by removing subjects of loss to follow-up (5,357), birth defects (254), medical abortions (2,476), induced labor (433), ectopic pregnancies (171), still births (574), preterm births (3,570), and non-singleton births (8,871). The selection process was further conducted by removing participants with unclear neonatal sex (15,326), extreme maternal age (<16 or >50) at delivery or missing (1,622), extreme pre-BMI (<12 or >50) or missing (6,688), unreported cigarette or alcohol consumption (1,521), unreported education levels (1,258) and unreported gestational week (5). The final 193,461 participants were included in our study. The distribution of PM_2.5_ over the entire pregnancy in 29 provinces of China was recorded in **Table S1** (two provinces were not listed due to small numbers involved in our study). There were 15 provinces and 50,328 (26.0%) participants correspondingly having their average PM_2.5_ lower than 60 μg/m^3^.

**Table 1** showed the baseline characteristics of pregnancy women and neonates included and excluded in our study. Male neonates (n=102,203) take the percentage of 52.8% of the population, which were more than female neonates (n=91,258). The mean duration of gestation was 39.25 weeks. The average birth weight of overall included newborns (n=193,461) was 3326.66 g. The overall average pre-pregnancy BMI was 21.04 kg/m^2^. The mean PM_2.5_ concentrations in each trimester and whole pregnancy were also calculated and the PM_2.5_ concentration in the third trimester was the highest among four periods. Other characteristics were also clearly shown in **Table 1**.

**Table 1:** Characteristics of the included and excluded pregnant women and neonates.

Characteristics	Number or mean	% or SD
Neonate’s sex		
Male	102,203	52.8%
Female	91,258	47.2%
Gestational week	39.25	1.45
Birthweight (g)	3326.66	514.23
Pre-pregnancy BMI ^a^ (kg/m^2^)	21.04	2.61
PM_2.5_ (μg/m^3^)		
First trimester	71.09	29.13
Second trimester	71.85	29.65
Third trimester	81.47	32.75
Whole pregnancy	75.33	22.57
Maternal age (year)	25.23	3.93
Smoking during pregnancy		
Yes	728	0.4%
Quit	1,095	0.6%
Never	191,638	99.1%
Drinking during pregnancy		
Yes	1,181	0.6%
Quit	1,266	0.7%
Never	191,014	98.7%
Educational level		
Junior high school or below	137,737	71.2%
Senior high school	37,015	19.1%
College or higher	18,709	9.7%
Prolonged pregnancy		
No	188,825	97.6%
Yes	4,636	2.4%
Multiparity		
No	152,584	78.9%
Yes	40,877	21.1%
Pre-pregnancy diabetes mellitus		
No	193,440	100.0%
Yes	21	0.0%
Pre-pregnancy hypertension		
No	193,363	99.9%
Yes	98	0.1%
Season of delivery		
Spring	52,053	26.9%
Summer	30,288	15.7%
Autumn	49,900	25.8%
Winter	61220	31.6%

a BMI, body mass index.

**Figure 2:**
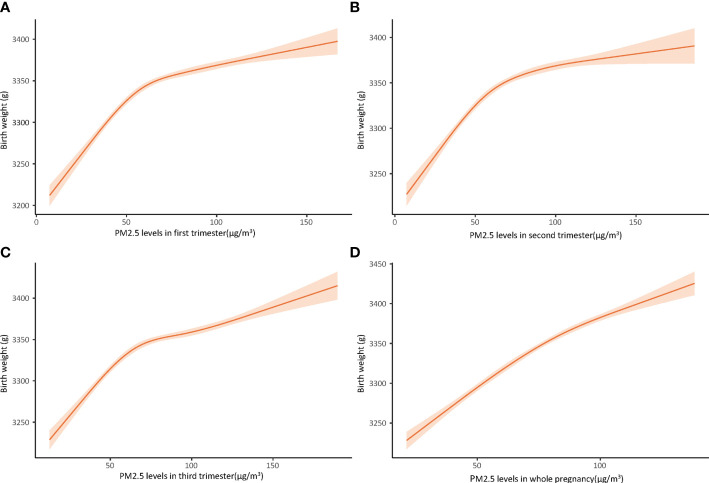
Dose-response relationship between PM_2.5_ concentration and birth weight in first trimester **(A)**, second trimester **(B)**, third trimester **(C)**, and whole pregnancy **(D)**, using cubic restricted model, adjusted for maternal age at delivery, neonatal sex, smoking during pregnancy, drinking during pregnancy, gestational week, pre-pregnancy BMI, educational level, prolonged pregnancy, multiparity, pre-pregnancy diabetes mellitus, pre-pregnancy hypertension, seasons.

Both unadjusted and adjusted multivariable linear regression models presented in **Table 2** indicated that PM_2.5_ was positively associated with birthweight. After adjusting for all confounding factors, every 10 μg/m^3^ increase in trimester-specific exposure to PM_2.5_ on birthweight was associated with increases in birthweight of 14.93 g (95%CI: 13.96, 15.89), 13.75 g(95%CI: 12.81, 14.69), and 8.79 g (95% CI: 8.09, 9.49) in the first, second, and third trimesters respectively. The newborn birthweight gained 17.15 g (95% CI: 16.15, 18.17) for every 10 μg/m^3^ rise of PM_2.5_ exposure throughout the entire pregnancy. Restricted cubic spline curves (**Figure 2**) illustrated nonlinear relationships between increase in birth weight and PM_2.5_. Curves for the first (A), second (B), and third trimesters (C) showed almost the same changing patterns, rising to a plateau at 50-60 μg/m^3^ of PM_2.5_ and keeping stable afterward. Variations at the terminal of the curves were considerable. However, the dose-response curve between PM_2.5_ and birthweight across the entire pregnancy (D) showed a nearly linear growth.

**Table 2:** Linear regression between PM_2.5_ concentration and birth weight ^a^.

PM_2.5_ concentration (μg/m^3^)	Unadjusted		Adjusted	
	β (95% CI)	P value	β (95% CI)	P value
First trimester	1.001 (0.923,1.080)	<0.001	1.493 (1.396,1.589)	<0.001
Second trimester	0.872 (0.795,0.950)	<0.001	1.375 (1.281,1.469)	<0.001
Third trimester	0.870 (0.800,0.940)	<0.001	0.879 (0.809,0.949)	<0.001
Whole pregnancy	1.697 (1.596,1.798)	<0.001	1.715 (1.615,1.817)	<0.001

a Adjusted for maternal age at delivery, neonatal sex, smoking during pregnancy, drinking during pregnancy, gestational week, pre-pregnancy BMI, educational level, prolonged pregnancy, multiparity, pre‐pregnancy diabetes mellitus, pre‐pregnancy hypertension, seasons.

**Figure 3:**
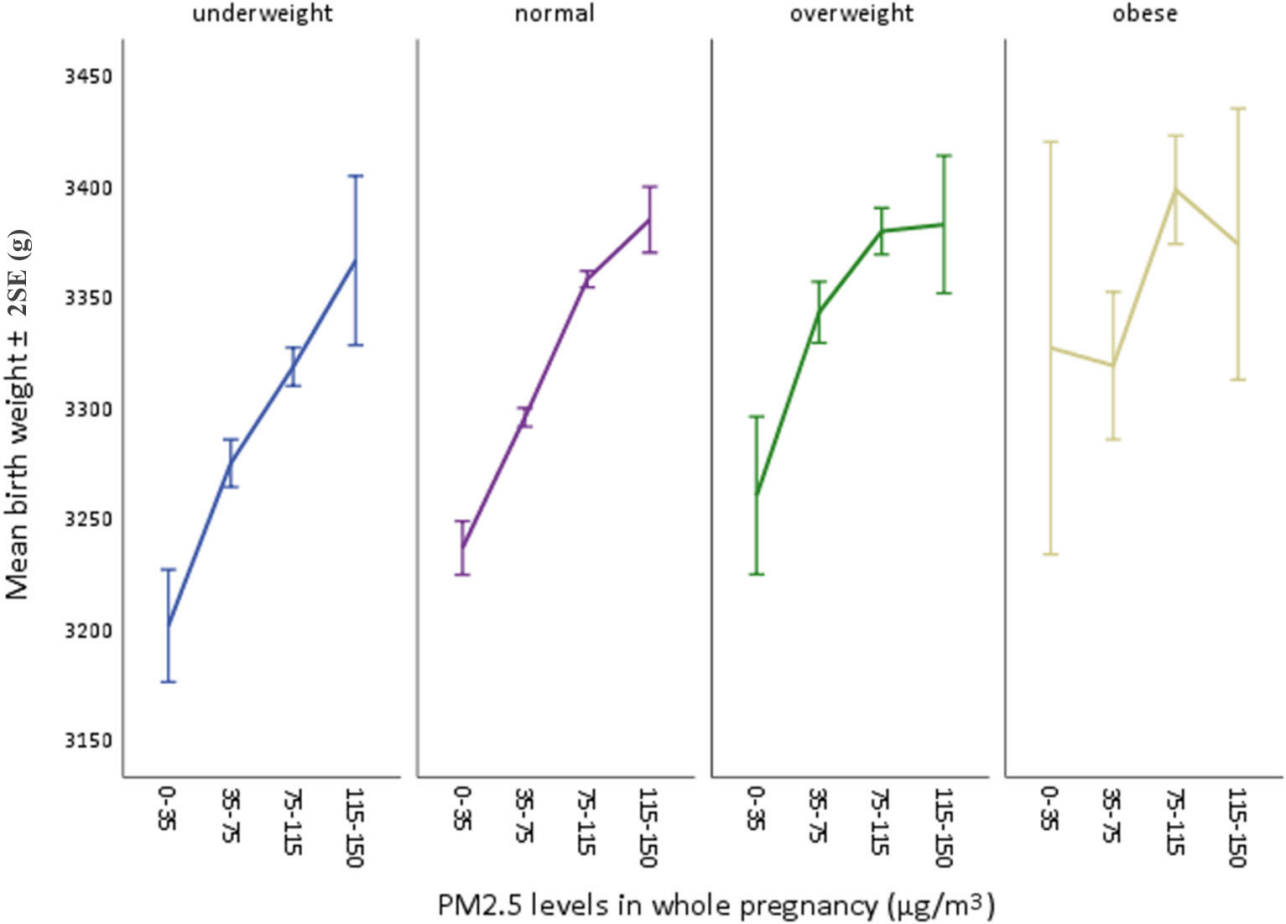
Mean birthweights by each PM_2.5_ level in four BMI subgroups. Four PM_2.5_ levels were: 0-35 (Chinese guideline II), 35-75 (mild), 75-115 (moderate), 115-150 (severe).

Multivariable linear regressions were also performed between pre-pregnancy BMI and birthweight with adjusted β coefficients 7.219 (95% CI: 6.342, 8.096) and adjusted β coefficient 7.012 (95% CI: 6.121, 7.902) (**Table 3**). Subgroup analysis was conducted in BMI-stratified subgroups (baseline characteristics shown in **Table S2** (**supplementary material**), which were classified as underweight (n=25,821), normal (n=146,081), overweight (n=18,187), obese (n=3,372). PM_2.5_ was divided into four specific groups with an interval of about 40 μg/m^3^ based on the air quality index (AQI) derived from a four-year (January 2015-December 2018) EAQ data of Eastern China (EC) (9). In each subgroup, mean values (± 2SE, standard error) of birthweight were calculated at four PM_2.5_ groups of the whole pregnancy and demonstrated in a broken-line graph (**Figure 3**). Each subgroup presented a generally increasing trend of mean birth weight as PM_2.5_ levels rose, and such an increasing pattern was less obvious and even turned to decrease as the BMI levels reached “overweight” and finally “obese”. It was also clearly observed that variations increased as sample size of subgroups decreased, suggesting less convincing patterns in small-sized groups. Therefore, to examine a potential modifying effect of pre-pregnancy BMI on the association between PM_2.5_ and birthweight, an interaction analysis was further conducted.

**Table 3:** Linear regression between pre-pregnancy BMI (kg/m^2^) and birthweight ^a^.

Unadjusted		Adjusted	
β (95% CI)	P value	β (95% CI)	P value
7.219 (6.342, 8.096)	<0.001	7.012 (6.121, 7.902)	<0.001

a Adjusted for maternal age at delivery, neonatal sex, smoking during pregnancy, drinking during pregnancy, gestational week, educational level, prolonged pregnancy, multiparity, pre‐pregnancy diabetes mellitus, pre‐pregnancy hypertension, seasons.

**Figure 4:**
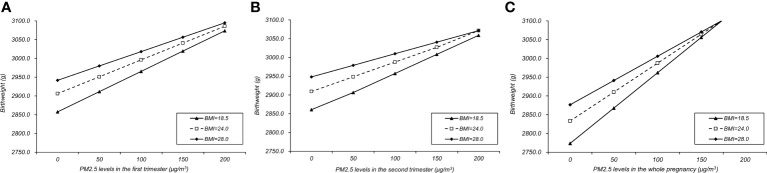
Interaction effects between PM_2.5_ and pre-pregnancy BMI in the first **(A)**, second **(B)** trimesters and the whole pregnancy **(C)**.

**Table 4** showed the interaction between pre-pregnancy BMI and PM_2.5_ exposure in three trimesters and the whole pregnancy on the increase of birth weight. In the first trimester, the interaction effect was statistically significant between exposure to PM_2.5_ and pre-pregnancy BMI (β=-0.033; 95% CI, -0.048, -0.018; p=0.0.030), showing a negative interaction in which PM_2.5_ might have less increasing impact on birthweight affected by different levels of pre-pregnancy BMI. Similar interactions could also be seen in both second trimester (P<0.001) and whole pregnancy (P=0.001), while the third trimester showed no significant interaction (p=0.329). The interaction effects were shown in **Figure 4**. The slopes of regression lines decreased as BMI increased from 18.5 to 28.0, illustrating negative interactions in the first, second trimesters and the whole pregnancy. In sensitivity analysis including 192,326 women and neonates whose birthweight ranged between 530 and 4970 kg/m^3^, the results were consistent with the main findings (**Tables S3, 4**).

**Table 4:** Interaction between PM_2.5_ (μg/m^3^) and pre-pregnancy BMI (kg/m^2^) on birthweight (g).

	Variable	β (95% CI) ^a^	P
First trimester	BMI	6.502 (6.049, 6.955)	<0.001
	PM_2.5_	0.996 (0.956, 1.036)	<0.001
	BMI*PM_2.5_^b^	-0.033 (-0.048, -0.018)	0.030
Second trimester	BMI	6.721 (6.266, 7.176)	<0.001
	PM_2.5_	0.910 (0.051, 1.310)	<0.001
	BMI*PM_2.5_^b^	-0.042 (-0.057, -0.027)	0.004
Third trimester	BMI	6.703 (5.814, 7.592)	<0.001
	PM_2.5_	0.880 (0.810, 9.500)	<0.001
	BMI*PM_2.5_^b^	-0.013 (-0.039, 0.013)	0.329
Whole pregnancy	BMI	6.164 (5.271, 7.056)	<0.001
	PM_2.5_	1.725 (1.623, 1.827)	<0.001
	BMI*PM_2.5_^b^	-0.062 (-0.099, -0.024)	0.001

^a^ Adjusted for maternal age at delivery, neonatal sex, smoking during pregnancy, drinking during pregnancy, gestational week, educational level, prolonged pregnancy, multiparity, pre‐pregnancy diabetes mellitus, pre‐pregnancy hypertension, seasons. ^b^ Interaction centralized: x’=x-μ

## Discussion

Our study innovatively explored the effect of intrauterine environment on neonatal birthweight from both the metabolic and physical environmental factors. An interaction between pre-pregnancy BMI and PM_2.5_ was discovered in the first and second trimesters as well as the whole pregnancy. We also found a nonlinear relationship between PM_2.5_ and birthweight increase, confirming the positive correlation between PM_2.5_ exposure and fetal weight gain. Over 190,000 pregnant women were included in our study, and the wide distribution of participants across the country provided good external validity.

First, the positive association between gestational PM_2.5_ and the neonatal birthweight denoted that exposure to PM_2.5_ during pregnancy related to higher risks of high birthweight and LGA. It should be noticed that birthweight increase should not be equal to better outcomes and high birthweight entailed higher risks of mortality and morbidity (35). The wide range of monthly-average PM_2.5_ in our study could cover from 0 to 210 μg/m^3^, while PM_2.5_ levels in many developed countries as well as some developing countries were far less than that in China (24, 25, 37). The restricted cubic spline curves generally presented an increasing trend, although appeared to reach a plateau at about 50-60 μg/m^3^ of PM_2.5_ (at the mild level). The curves implied that birthweight was more sensitive to slight or mild PM_2.5_ pollution, and pregnant women should be alert even at a slightly mild level of air pollution. It was noticed that PM_2.5_ exposure was highest in the third trimester. Seasonality is an important factor in which heavy PM2.5 usually occurs during more polluted seasons such as winter (19). Our data presented a higher percentage of neonates born in winter, which could partially explain a significant increase of PM_2.5_ exposure in the third trimester. A similar increasing effect of pre-pregnancy BMI on birthweight was also found in three trimesters. A study by Yu et al. found that pre-pregnancy overweight or obese increased the risk of LGA (OR=1.53; 95%, 1.44-1.63), high birthweight (OR=2.00; 95% CI, 1.84-2.18), macrosomia (OR=1.67; 95% 1.42-1.97), and probably subsequent offspring overweight or obese (38). In addition, maternal weight gain during pregnancy was found as a mediator between pre-pregnancy BMI and birthweight increase (39).

In our study, a negative interaction effect was unprecedentedly established between gestational PM_2.5_ exposure and pre-pregnancy BMI on neonatal birthweight in the first, second trimesters and the whole pregnancy, in which pre-pregnancy BMI compromised the increasing effect of PM_2.5_ on birthweight. Pregnancy is a well-known stage of susceptibility in oxidative stress, mainly produced by a normal systemic inflammatory response (40). Obesity is characterized by chronic inflammation along with many other metabolic syndromes such as diabetes mellitus and hypertension (11). The interpretation of such a negative interaction can be put in two aspects: the lifestyle and biological factors. It is well-known that obesity is related to an individual’s living habits. Overweight or obese people may have unhealthy lifestyle habits such as overeating and less outdoor exercise, and the amount of outdoor exercise directly affects the amount of PM2.5 exposure. Therefore, we speculate that overweight and obese individuals have reduced PM2.5 exposure by spending less time outdoors compared to normal weight, thereby weakening the original role of PM2.5 in increasing birthweight. For the biological factors, it has been discovered that maternal metabolic environment affects ovum production, gene expression of zygote and placental development during pregnancy (8). Obesity increases the susceptibility of environmental pollution during the pregnancy mainly by epigenetic inheritance in fetus (41). Studies have shown that the activity of insulin-sensitive genes in obese pregnant women is significantly down-regulated in early pregnancy, leading to elevated insulin levels (42). High insulin levels enhance the activity of the IGF-1 pathway in obese pregnant women and affect fetal growth and development through the placenta, increasing the risk of fetal overweight (43). Elevated concentrations of TNF-α, IL-1b, IL-6 and leptin may also worsen insulin resistance and increase fetal overgrowth (44). Similarly, the mechanism of how PM2.5 affects fetal weight can also attribute to epigenetic inheritance. Recent studies have found that PM2.5 may affect fetal growth and development by changing fetal IGF2 gene expression level (41). IGF-2 is a growth factor in homology with IGF-1, which is expressed by an imprinted gene on paternal chromosome 11 (45). The loss of paternalistic imprinting leads to IGF-2 overexpression and fetal overgrowth (45). IGF-1 receptor (IGF1R) is widely expressed in different tissues and both insulin, IGF-1 and IGF-2 can bind to IFG1R to activate downstream signal transduction, while IGH-2 receptor can only be specifically bound by IGF-2 (46). Based on these evidence, we conclude that obese women with pre-pregnancy BMI may increase maternal and placental deposition of metabolites such as lipids through high levels of IGF-1, and this strong effect may cover up the PM_2.5_’s effect on IGF-2 gene expression. However, the mechanism of environmental factors and metabolic factors on fetal growth and development is rather complex. Further research is necessary to explore the mechanisms of biological interaction during the pregnancy. It is noteworthy that there is no interaction found in the third trimester, which should be the crucial period of fetal growth and least affected by genetic factors. These mechanisms suggest that such an interaction may interfere the early and middle stage of pregnancy by mainly affecting the regulation of epigenetics and growth factors and thus play a profound role in late pregnancy as well as postnatal growth.

This is the first analysis to our knowledge of the potential interaction between pre-pregnancy BMI and gestational PM_2.5_ in a large Chinese cohort study. Interactions between pre-pregnancy BMI and many other factors were also discovered by previous studies. An interaction between pre-pregnancy BMI and gestational passive smoking was found related to macrosomia and LGA (47) and the effect of smoking during pregnancy on SGA and birth weight was markedly reduced among obese and overweight women (48). Interactive effects between different types of metabolic disorders were also assessed. Pre-pregnancy overweight/obesity and hypertensive disorders jointly increasing the risks of obesity and hypertension in offspring or throughout their life course (28, 49). In our study, the negative interaction effect implied a biological interaction between *in vivo* and *in vitro* factors, which could be conceptualized in one of two ways: PM_2.5_ might dampen BMI’s effect on birthweight, and/or higher pre-pregnancy BMI might attenuate PM_2.5_’s effects on birthweight. As for the contradictions of opposite adverse outcomes of PM_2.5_ on birthweight in different studies, we suppose that different metabolic status between different races and lifestyles, especially maternal overweight/obesity, can interfere the consistency in how PM_2.5_ affects birthweight. However, the detailed mechanisms remained unclear.

Our study has some strengths and several limitations. First, a large nationwide cohort study was conducted aiming to find a potential interaction between air pollution and metabolic status on birthweight. Another strength was to contradict the conventional opinion that pre-pregnancy BMI and air pollutions jointly had additive effect on birthweight (11), which shed light on future studies. In addition, to our current knowledge, this is the first study on a large Chinese cohort exploring the biological interaction between pre-pregnancy BMI and PM_2.5_ on birthweight, linking the metabolic status and environmental stressors in a combined manner. Furthermore, our study also provided a reasonable assumption that such an interaction may interfere fetal growth signal pathways. One of the limitations of our study was that we applied the estimated PM_2.5_ concentration instead of the ground monitored levels as the result of no ground monitoring station had been established until 2013. Another limitation of our study was the lacks of records of the actual residence of pregnant women. Although we ruled out individuals whose labor places were not coincident with their registered residence, mobility could still probably exist. We suggest that the public and researchers pay more attention to the potential risk of the complex relationship between air pollution and metabolic abnormalities on pregnancy outcomes, and hope to provide some enlightenment for future research.

## Conclusion

Both maternal exposure to PM_2.5_ during pregnancy and pre-pregnancy BMI have positive associations with birth weight throughout pregnancy. More importantly, our findings indicate a negative interaction between pre-pregnancy BMI and PM_2.5_ on birthweight in the first, second trimesters and the whole pregnancy. Further experiments and researches are necessary to identify the biological mechanism of interaction.

## Data Availability Statement

The data analyzed in this study is subject to the following licenses/restrictions: Our research data were derived from the National Free Preconception Health Examination Project (NFPHEP). Requests to access these datasets should be directed to HP, PanHui@pumch.cn.

## Ethics Statement

This study was reviewed and approved by Institutional Review Board of the National Research Institute for Family Planning, Beijing, China. The patients/participants provided their written informed consent to participate in this study. Written informed consent was obtained from the individual(s) for the publication of any potentially identifiable images or data included in this article.

## Author Contributions

HD, YS, YZ, SW, HZ, SC, and HPcontributed to the study concept and design. HD drafted the work. YZ has made substantial contributions to the acquisition, analysis, or interpretation of data for the work. YS conducted the statistical analysis. HD and YS analyzed the results. YS wrote the first draft. HD and SC provided editing and writing assistance for important intellectual content. HD, YS, and SC finalized the manuscript. All authors contributed to the article and approved the submitted version.

## Acknowledgments

This study was supported by the CAMS Initiative for Innovative Medicine (grant no. 2016-I2M-1-008), Health Science Promotion Project of Beijing (grant no. 2018-TG-35), Beijing Municipal Natural Science Foundation (grant no. 7192153), and National Natural Science Foundation of China (grant no. 81673184).

## Conflict of Interest

The authors declare that they do not have any commercial or associative interest that represents a conflict of interest in connection with the work submitted.

## Publisher’s Note

All claims expressed in this article are solely those of the authors and do not necessarily represent those of their affiliated organizations, or those of the publisher, the editors and the reviewers. Any product that may be evaluated in this article, or claim that may be made by its manufacturer, is not guaranteed or endorsed by the publisher.
