# Supplementary material for: Interaction of PM2.5 and pre-pregnancy body mass index on birth weight: A nationwide prospective cohort study
Source: Front Endocrinol (Lausanne). 2022 Jul 26;13:963827. doi: 10.3389/fendo.2022.963827 (PMC9360486; doi:10.3389/fendo.2022.963827)
Supplement: Supplementary file 1 [file DataSheet_1.pdf]

Supplementary Material

1 Supplementary Tables

TABLE S1 Spacial distribution of PM2.5. Distribution of exposure to PM2.5 over the entire pregnancy in each province in China based on data from experimental counties.

Province	n	Mean PM2.5 (μg/m^3) ± SD	Province	n	Mean PM2.5 (μg/m^3) ± SD
Tianjin	158	103.87 ± 5.04	Jiangxi	2781	60.19 ± 7.54
Hebei	16530	102.60 ± 18.72	Liaoning	122	59.54 ± 8.21
Henan	20343	99.79 ± 12.18	Qinghai	82	59.10 ± 3.86
Chongqing	7081	91.47 ± 9.56	Guizhou	1935	57.06 ± 5.85
Shandong	5565	89.33 ± 14.55	Guangxi	5376	56.92 ± 8.91
Sichuan	6403	87.27 ± 11.37	Jilin	3887	56.17 ± 4.77
Beijing	265	86.32 ± 10.15	Gansu	3793	55.93 ± 5.88
Hubei	36732	83.23 ± 10.73	Xinjiang	719	53.43 ± 17.48
Anhui	3617	81.68 ± 9.93	Inner Mongolia	211	51.84 ± 12.12
Jiangsu	11743	77.37 ±6.73	Guangdong	21175	48.48 ± 10.21
Shanxi	820	72.86 ± 7.07	Ningxia	286	45.09 ± 3.85
Hunan	24718	72.86 ± 7.31	Heilongjiang	391	39.51 ± 4.41
Zhejiang	884	63.98 ± 7.30	Fujian	793	38.28 ± 7.91
Shaanxi	7795	61.66 ± 11.99	Yunnan	8700	28.36 ± 3.52
			Hainan	67	25.07 ± 6.13

TABLE S2 Mean values of BMI, PM2.5 and birth weight divided by BMI subgroups

	Underweight <=18.4 kg/m^2	Normal 18.5-23.9 kg/m^2	Overweight 24.0-27.9 kg/m^2	Obese >=28.0 kg/m^2	Maximum standardized difference [a]
Proportion of participants (%)	25821 (13.3%)	146081 (75.5%)	18187 (9.4%)	3372 (1.7%)	/
Neonate's sex					
Male	13367 (51.8%)	77384 (53.0%)	9685 (53.3%)	1767 (52.4%)	0.03
Female	12454 (48.2%)	68697 (47.0%)	8502 (46.7%)	1605 (47.6%)	
Maternal age (year)	24.43 ± 3.25	25.20 ± 3.89	26.26 ± 4.61	26.95 ± 4.76	0.62
Gestational week	39.20 ± 1.43	39.26 ± 1.44	39.24 ± 1.50	39.23 ± 1.52	0.04
Educational level					
Junior high school or below	16667 (64.5%)	104201 (71.3%)	14144 (77.8%)	2725 (80.8%)	0.37

Senior high school	5688 (22.0%)	28009 (19.2%)	2837 (15.6%)	481 (14.3%)	0.20
College or higher	3466 (13.4%)	13871 (9.5%)	1206 (6.6%)	166 (4.9%)	0.30
Smoking during pregnancy					
Yes	94 (0.4%)	557 (0.4%)	67 (0.4%)	10 (0.3%)	0.03
Quit	119 (0.5%)	838 (0.6%)	121 (0.7%)	17 (0.5%)	
Never	25608 (99.2%)	144686 (99.0%)	17999 (99.0%)	3345 (99.2%)	
Drinking during pregnancy					0.01
Yes	160 (0.6%)	885 (0.6%)	118 (0.6%)	18 (0.5%)	
Quit	137 (0.5%)	970 (0.7%)	142 (0.8%)	17 (0.5%)	
Never	25524 (98.8%)	144226 (98.7%)	17927 (98.6%)	3337 (99.0%)	
Birth weight (g)	3296.48 ± 515.55	3326.70 ± 510.97	3361.39 ± 531.17	3368.44 ± 536.77	0.13
PM2.5 (μg/m^3)					
First trimester	69.42 ± 28.07	70.71 ± 29.15	75.28 ± 29.64	77.80 ± 30.81	0.28
Second trimester	72.18 ± 28.97	71.03 ± 29.51	76.47 ± 30.58	79.96 ± 32.02	0.29
Third trimester	82.59 ± 32.05	80.52 ± 32.63	86.12 ± 33.56	89.31 ± 34.98	0.26
Whole pregnancy	75.35 ± 21.63	74.60 ±22.47	79.83 ± 23.40	82.88 ± 25.02	0.35
Multiparity					
No	20940 (81.1%)	116631 (79.8%)	12782 (70.3%)	2231 (66.2%)	0.34
Yes	4881 (18.9%)	29450 (20.2%)	5405 (29.7%)	1141 (33.8%)	
Pre-pregnancy BMI (kg/m^2)	17.63 ± 0.73	20.88 ± 1.39	25.33 ± 1.08	30.82 ± 3.46	5.28
Prolonged pregnancy					
No	25233 (97.7%)	142663 (97.7%)	17652 (97.1%)	3277 (97.2%)	0.03
Yes	588 (2.3%)	3418 (2.3%)	535 (2.9%)	95 (2.8%)	
Pre-pregnancy diabetes mellitus					
No	25820 (100.0%)	146069 (100.0%)	18182 (100.0%)	3369 (99.9%)	0.04
Yes	1 (0.0%)	12 (0.0%)	5 (0.0%)	3 (0.1%)	
Pre-pregnancy hypertension					
No	25814 (100.0%)	146031 (100.0%)	18164 (99.9%)	3354 (99.5%)	0.10

Yes	7 (0.0%)	50 (0.0%)	23 (0.1%)	18 (0.5%)	
Season of delivery					
Spring	7481 (29.0%)	38608 (26.4%)	4987 (27.4%)	977 (29.0%)	0.06
Summer	3611 (14.0%)	23263 (15.9%)	2896 (15.9%)	518 (15.4%)	0.05
Autumn	6199 (24.0%)	38236 (26.2%)	4621 (25.4%)	844 (25.0%)	0.05
Winter	8530 (33.0%)	45974 (31.5%)	5683 (31.2%)	1033 (30.6%)	0.05

[a] Standardized difference greater than 0.1 was considered significantly different.

TABLE S3 Linear regression between $PM_{2.5}$ concentration and birth weight [a]

PM2.5 concentration ($\mu g/m^3$)	Unadjusted		Adjusted	
	β (95% CI)	P value	β (95% CI)	P value
First trimester	1.088 (1.015, 1.161)	<0.001	1.611 (1.523, 1.700)	<0.001
Second trimester	0.917 (0.845, 0.988)	<0.001	1.471 (1.384, 1.558)	<0.001
Third trimester	0.961 (0.896, 1.026)	<0.001	1.373 (1.294, 1.452)	<0.001
Whole pregnancy	1.839 (1.745, 1.933)	<0.001	1.934 (1.837, 2.030)	<0.001

[a] Adjusted for maternal age at delivery, neonatal sex, smoking during pregnancy, drinking during pregnancy, gestational week, pre-pregnancy BMI, educational level, prolonged pregnancy, multiparity, pre-pregnancy diabetes mellitus, pre-pregnancy hypertension, seasons.

TABLE S4 Interaction between $PM_{2.5}$ ($\mu g/m^3$) and pre-pregnancy BMI (kg/m^2) on birthweight (g).

	Variable	β (95% CI) [a]	P
First trimester	BMI	9.158 (6.983, 11.333)	<0.001
	PM2.5	2.430 (1.842, 3.019)	<0.001
	BMI*PM2.5 [b]	-0.039 (-0.066, -0.011)	0.006
Second trimester	BMI	10.462 (8.293, 12.630)	<0.001
	PM2.5	2.592 (2.021, 3.163)	<0.001
	BMI*PM2.5 [b]	-0.053 (-0.080, -0.026)	<0.001
Third trimester	BMI	8.451 (6.236, 10.666)	<0.001
	PM2.5	1.872 (1.354, 2.391)	<0.001
	BMI*PM2.5 [b]	-0.024 (-0.048, 0.001)	0.056
Whole pregnancy	BMI	11.676 (8.843, 14.509)	<0.001
	PM2.5	3.443 (2.703, 4.182)	<0.001
	BMI*PM2.5 [b]	-0.071 (-0.106, -0.037)	<0.001

[a] Adjusted for maternal age at delivery, neonatal sex, smoking during pregnancy, drinking during pregnancy, gestational week, pre-pregnancy BMI, educational level, prolonged pregnancy, multiparity, pre-pregnancy diabetes mellitus, pre-pregnancy hypertension, seasons.
